# A Pharmacogenetically Guided Acenocoumarol Dosing Algorithm for Chilean Patients: A Discovery Cohort Study

**DOI:** 10.3389/fphar.2020.00325

**Published:** 2020-04-06

**Authors:** Angela Roco, Elena Nieto, Marcelo Suárez, Mario Rojo, Maria Paz Bertoglia, Gabriel Verón, Francisca Tamayo, Annabella Arredondo, Daniela Cruz, Jessica Muñoz, Gabriela Bravo, Patricio Salas, Fanny Mejías, Gerald Godoy, Paulo Véliz, Luis Abel Quiñones

**Affiliations:** ^1^Laboratory of Chemical Carcinogenesis and Pharmacogenetics, Department of Basic and Clinical Oncology, Faculty of Medicine, University of Chile, Santiago, Chile; ^2^Escuela de Bioquímica Facultad de Ciencias de la Vida, Universidad Andrés Bello, Santiago, Chile; ^3^Western Metropolitan Health Service, Santiago, Chile; ^4^San Juan de Dios Hospital, Santiago, Chile; ^5^Latin American Network for Implementation and Validation of Clinical Pharmacogenomics Guidelines (RELIVAF-CYTED), Madrid, Spain; ^6^Institute of Population Health, University of Chile, Santiago, Chile; ^7^Instituto de Salud Pública, Universidad Andrés Bello, Santiago, Chile; ^8^Curacaví Hospital, Curacaví, Chile; ^9^Dr. Salvador Allende G. Reference Health Center, Santiago, Chile; ^10^San José de Melipilla Hospital, Melipilla, Chile

**Keywords:** acenocoumarol, coumarins, algorithm, pharmacogenetics, pharmacogenomics, anticoagulation

## Abstract

**Background:**

Vitamin K antagonists (VKA) are used as prophylaxis for thromboembolic events in patients with cardiovascular diseases. The most common VKA are warfarin and acenocoumarol. These drugs have a narrow therapeutic margin and high inter-individual response variability due to clinical and pharmacogenetic variables.

**Objective:**

The authors aim to develop an algorithm comprised of clinical and genetic factors to explain the variability in the therapeutic dose of acenocoumarol among Chilean patients

**Methodology:**

DNA was obtained from 304 patients as a discovery cohort with an international normalized ratio (INR) range of 2.0–3.0. The non-genetic (demographic and clinical) variables were also recorded. Genotype analyses were performed using real-time PCR for *VKORC1* (*rs9923231*), *VKORC1* (*rs7294*), *GGCx* (*rs11676382*)*, CYP4F2* (*rs2108622*)*, ABCB1* (*rs1045642*), CYP2C9*2 (*rs1799853*), *ApoE* (*rs429358*), and CYP2C9*3 (*rs1057910*).

**Results:**

The clinical variables that significantly influenced the weekly therapeutic dose of VKA were age, sex, body mass index (BMI), and initial INR, collectively accounting for 19% of the variability, and the genetic variables with a significant impact were *VKORC1* (*rs9923231*), CYP2C9*2 (*rs1799853*), and CYP2C9*3 (*rs1057910*), explaining for another 37% of the variability.

**Conclusion:**

We developed an algorithm that explains 49.99% of the variability in therapeutic VKA dosage in the Chilean population studied. Factors that significantly affected the dosage included *VKORC1*, CYP2C9*2, and CYP2C9*3 polymorphisms, as well as age, sex, BMI, and initial INR.

## Introduction

Cardiovascular diseases (CVD) have been highlighted as a health priority by several institutions worldwide, including the World Health Organization (WHO) through its Global Action Plan for the Prevention and Control of Non-communicable Diseases 2013–2020. Oral anticoagulants are indicated for patients who survive a cardiovascular disease in order to prevent new thromboembolic conditions (WHO, 2013).

Coumarin anticoagulants, also called vitamin K antagonists (VKA), include drugs such as warfarin, acenocoumarol, and phenprocoumon. VKA are highly effective antithrombotic agents used to prevent complications associated with atrial fibrillation, artificial heart valves, and thromboembolic diseases (such as deep venous thrombosis and pulmonary embolism). The most commonly used VKA are warfarin and acenocoumarol (Pengo et al., 2006; Guyatt et al., 2012; Verhoef et al., 2013; Quiñones et al., 2015; Nieto et al., 2019).

To calibrate weekly VKA doses, physicians use prothrombin time [expressed as international normalized ratio (INR) value] empirically to make adjustments at each visit until the patient reaches therapeutic range. Time in therapeutic range (TTR) is then used to assess the quality of anticoagulation. TTR should be above 65% to protect the patient against thrombotic or hemorrhagic risk, according to the National Institute for Health and Care Excellence (NICE) guidelines, or above 70%, according to the European Consensus (NICE, 2014; Esteve-Pastor et al., 2018). Major bleeding is the most concerning adverse effect of anticoagulant therapy, but the risk of bleeding associated with VKA is difficult to estimate. Risk estimates vary according to study design, with an approximate annual incidence of 0.6% for fatal hemorrhage, 3% for major hemorrhage, 9.6% for minor hemorrhage, and 0.2–0.4% for intracranial bleeding depending on the series (Ageno et al., 2012; Fitzmaurice et al., 2016; Haas et al., 2016; Bosch et al., 2017).

Few studies have characterized Chilean patients treated with coumarin derivatives; however, the GARFIELD-AF study, which assessed Chilean patients with atrial fibrillation (AF), reported in 2017 that the median TTR was 40% for 971 patients treated in several public hospitals and private clinics. In that sample, 36 patients (3.6%) had a cerebrovascular accident as an adverse event (Corbalán et al., 2017). According to the same study, the average number of days to reach the desired therapeutic range was 301.6 days, with a median of 204 days, among the patients treated at the Western Metropolitan Health Service (WMHS) in Santiago, Chile. The physicians at the facilities studied relied exclusively on INR values for dose adjustment. Furthermore, the median TTR was only 50% in these AF patients; this low value is worrisome as these patients are at an elevated risk of a new thrombotic pattern or a hemorrhagic complication while out of the therapeutic range (Nieto et al., 2019).

In recent decades, pharmacogenetic research has addressed the relationship between the genetic factors and the required doses of VKA. The most-studied polymorphisms include *CYP2C9*, *VKORC1*, *CYP4F2, GGCx*, and *ABCB1* (Leschziner et al., 2007; Caldwell et al., 2008; De Oliveira Almeida et al., 2011; Johnson et al., 2011; Sun et al., 2015; Shendre et al., 2016). Several algorithms with genetic and non-genetic variables have been developed to calculate VKA dosage, improving the efficacy and safety of the treatment according to TTR results (Wu et al., 2008; Ramos et al., 2012; Borobia et al., 2012; Pirmohamed et al., 2013; Wypasek et al., 2014; Santos et al., 2015; Johnson et al., 2017; Galvez et al., 2018). These algorithms seem to be more precise when developed and applied in specific populations as the frequency of the polymorphisms described depends on ethnicity. Furthermore, consumption of green vegetables, which are rich in vitamin K, also varies by geographical location (Visser et al., 2005; Kocael et al., 2019).

Several algorithms have been published for acenocoumarol in diverse populations. Verde et al. (2010) constructed an “acenocoumarol-dose genotype score” based on the number of alleles associated with a higher required acenocoumarol dosage carried by each patient for each polymorphism. In addition, two algorithms that include demographic, clinical, and genetic factors have been published for Indian populations, with coefficients of determination of 41 and 61.5% (Rathore et al., 2012; Krishna-Kumar et al., 2015).

Two algorithms have been developed for European populations. The first, designed for a mixed European population, includes *CYP2C9* and *VKORC1* polymorphisms and clinical variables (age, sex, weight, height, and amiodarone use). The genetic components in the algorithm explained 52.6% of the dosage variance, and the non-genetic variables explained 23.7% (van Schie et al., 2011). The second algorithm was developed in a cohort of 973 patients undergoing anticoagulation therapy and includes clinical factors [age and body mass index (BMI)] and genetic variants (*VKORC1*, *CYP2C9*, and *CYP4F2* polymorphisms). The genetic and clinical variables explained 50 and 16% of the variance in acenocoumarol dosage, respectively (Cerezo-Manchado et al., 2013).

The aim of this study was to generate a preliminary algorithm with clinical and genetic factors that explains the variability in the therapeutic dose of VKA in Chilean patients. In order to achieve that, we have investigated the association of relevant single nucleotide polymorphisms (SNPs) (**Table 1**) with acenocoumarol dosage.

**Table 1:** Genetic variants and their potential effect on vitamin K antagonists (VKA) dosage (modified from Visser et al., 2005; Kocael et al., 2019).

Enzyme	Gene	SNP	Change	Effect on VKA dose
MDR1	*ABCB1*	rs1045642	c.3435C > T, exon 26 p.Ile1145Ile silent	Decrease
CYP4F2	*CYP4F2*	rs2108622	c.1297 C > T, exon 11 p.Val433Met missense	Increase
CYP2C9	CYP2C9*2	rs1799853	c.3608C > T, exon 3 p.Arg144Cys missense	Decrease
	CYP2C9*3	rs1057910	c.42614 A > C, exon 7 p.Ile359Leu missense	Decrease
GGCx	*GGCX*	rs11676382	c.2084+45 C > G Intron 14	Decrease
VKORC1	*VKORC1*	rs9923231	-1639 G > A promotor	Decrease
	*VKORC1*	rs7294	3730 G > A 3′UTR	Increase
APOE	*ApoE*	rs429358	T > C, exon 4 p.Arg176Cys missense	Decrease

## Materials and Methods

### Study Design

A retrospective cohort study was carried out between March and December of 2018 among patients treated with acenocoumarol (Coarol, Andrómaco, Santiago, Chile) as an antithrombotic therapy at WMHS in the Santiago and Melipilla provinces of Chile. INR measurements were performed in a capillary sample using CoaguChek pro II^®^ equipment (Roche Mannheim, Germany). The sample size was determined according to the frequency of the carriers with the variant allele carriers in the population under study using PS Power and Sample Size Calculations Version 3.0, January 2009, considering 80% power, *α* = 0.05, OR = 2.0, and the less frequent minor allele frequencies (MAF) SNP CYP2C9*3 (rs1057910), according to literature (EMBL-EBI, 2019). The minimum patient number obtained was of 284 patients.

### Initial Dosage and Dose Adjustment and Frequency of INR Monitoring

The initial dose was one 4-mg tablet of acenocoumarol on day 1. On day 2, the dose was decreased to 50% (half a tablet). The INR [(PT^test^/PT^normal^)^ISI^] (Riley et al., 2000) was controlled on day 3; thus, if the INR was higher than 1.8, the dose was again reduced by 50%, and the patients were checked in 2 days for medical control to adjust the dose according to the INR results. The weekly therapeutic dose of acenocoumarol was modified at each control according to the INR value of the patient. The dosage for patients with INR ≤1.5 was increased by 20%, those of patients with INR >1.5– < 2.0 was increased by 5%, those of patients with INR >3.0–3.5 was decreased by 5%, those of patients with INR >3.5– < 6.0 had their dose discontinued and decreased by 15%, while patients with INR ≥6 had their dose suspended and controlled in 3 days to start again. For INR values within the therapeutic range, the patients were seen in 4 weeks for control (Ansell et al., 2008; Marcatto et al., 2018). Patients having three consecutive INR values in the therapeutic range (2.0–3.0) at the same dose of acenocoumarol were included in this study.

### Ethics Statement

The research was authorized by the Ethics Committees of the University of Chile Faculty of Medicine, Project 222-2015, and the WMHS, Protocol No. 027/2016.

### Patient Data

Data were obtained from clinical centers and managed with the statistical module of the anticoagulant treatment dosing software TAONet (Roche Diagnostics, Mannheim, Germany).

### Genotypic Analysis

Genomic DNA was isolated from the peripheral blood samples of the subjects using the High Pure PCR Template Preparation Kit (catalog number 11796828001; Roche Diagnostics GmbH, Mannheim, Germany). *VKORC1* (*rs9923231*), *VKORC1* (*rs7294*), *GGCx* (*rs11676382*), *CYP4F2* (*rs2108622*), *ApoE* (*rs429358*), *ABCB1* (*rs1045642*), CYP2C9*2 (*rs1799853*), and CYP2C9*3 (*rs1057910*) were analyzed using the *TaqMan*^®^ SNP Genotyping Assay (catalog number 4362691, Thermo Fisher Scientific, Waltham, MA, United States) in a Stratagene Mx3000p real-time PCR system. For quality assurance purposes, we randomly choose 20% of the samples for (a) repetition of the analysis and (b) PCR-RFLP analysis for coincidence. When the analyses were not coincident, we excluded the samples.

### Statistical Analyses

Data analysis was performed with STATA 15.0^®^ software. The Shapiro–Wilk test was used to determine whether the sample had a normal distribution for the continuous variable weekly therapeutic dose (WTD; mg/week), that is, the acenocoumarol dosage at which patients were in the therapeutic range. The ladder command from STATA 15.0^®^ was used to choose the best normal distribution expression. Finally, a linear regression analysis was performed among genetic (SNPs) and non-genetic variables with the logarithm of the WTD in the therapeutic range (2.0–3.0), incorporating adjustment variables (*p*-value > 0.05). The performance of the algorithm was evaluated by calculating the adjusted coefficient of determination (*R*^2^) that represents the variability explained by the model. Hardy–Weinberg equilibrium (HWE) was tested for all SNPs using chi^2^ test.

## Results

### Characteristics of the Study Population

**Figure 1:**
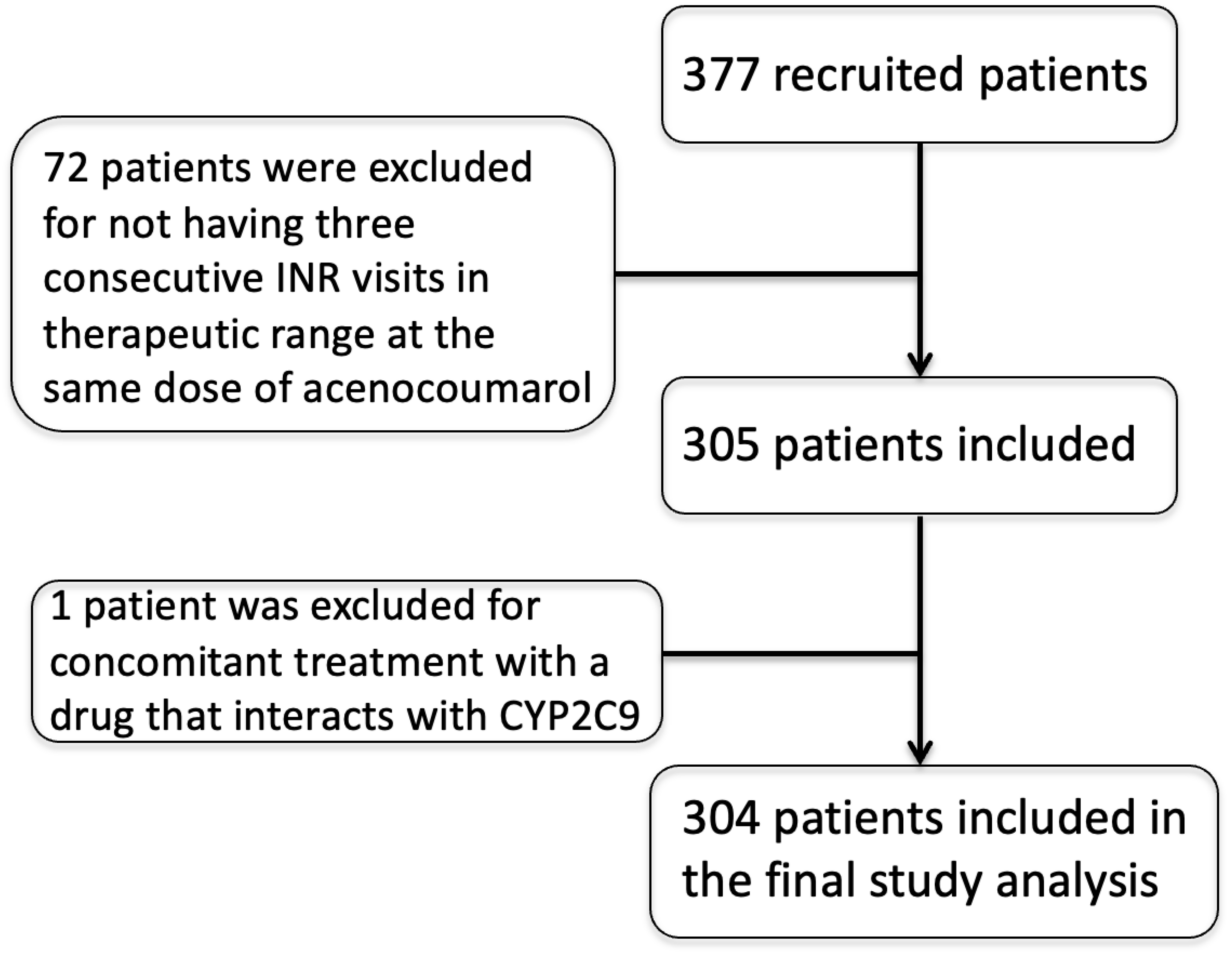
Patient recruitment and selection flowchart.

We enrolled 377 patients on oral anticoagulant treatment with acenocoumarol. A total of 72 patients were excluded for not having three consecutive INR values in the therapeutic range (2.0–3.0) at the same dose of acenocoumarol, and one was excluded for concomitant treatment with amiodarone (**Figure 1**). The final sample included 304 patients. As shown in **Table 2**, 47.4% of the patients were women, and 52.6% were men. The average age was 65.01 ± 13.99 years. The Caucasian–Amerindian admixture was 9.8% Amerindian and 90.2% Caucasian for this population (Acuña et al., 2000). No patient had bleeding nor myocardial infarction/stroke during the study.

**Table 2:** Characteristics of the study patients.

Characteristic	*N* (%)
Female	144 (47.4%)
Male	160 (52.6%)
Total	304 (100%)
% mixed Caucasian–aboriginal ethnicity	9.80%
Age ± SD [range] (years)	65.01 ± 13.99 [22–95]
Body mass index (BMI) (kg/m^2^) ± SD (median)	29.2 ± 5.7 (28.4)
INR range	2.0–3.0
Acenocoumarol	100%
Weekly therapeutic dose of acenocoumarol (mg/week) ± SD	14.6 ± 2.2
Acenocoumarol dosage, mg/week; range (median)	3.5–46 (13)
Time to reach therapeutic range (days); average ± SD	308 ± 343
Time to reach therapeutic range (days); range (median)	3–353 (206)
**Primary diagnosis**	*N* (%)
Rhythm disorder	156 (51.3%)
Venous thrombosis with/without pulmonary thromboembolism	64 (21.1%)
Occlusive arterial disease	24 (7.9%)
Stroke	17 (5.6%)
Others	43 (14.1%)
Total	304 (100%)
**Secondary diagnosis**	*N* (%)
Arterial hypertension	64 (25.6%)
Diabetes mellitus	27 (10.8%)
Cardiomyopathy	26 (10.4%)
Other	88 (42.9%)
Total	205 (100%)

SD, standard deviation.

### Genotype Distribution in the Study Population

The analysis of the HWE showed that only CYP2C9*3 (rs1057910) is not in HWE (chi^2^ = 4.67). All other SNPs, *VKORC1* (rs9923231) (chi^2^ = 0.09), *VKORC1* (rs7294) (chi^2^ = 0.62), GGCx (rs11676382) (chi^2^ = 1.2), *CYP4F2* (rs2108622) (chi^2^ = 1.02), *ApoE* (rs429358) (chi^2^ = 0.08), *ABCB1* (rs1045642) (chi^2^ = 0.68), and CYP2C9*2 (rs1799853) (chi^2^ = 2.33), are in HWE.

The MAF were as follows: 0.467 for *VKORC1* (rs9923231), 0.311 for *VKORC1* (rs7294), 0.229 for *CYP4F2* (rs2108622), 0.081 for CYP2C9*2 (rs1799853), 0.041 for CYP2C9*3 (rs1057910), 0.036 for *GGCx* (rs11676382), 0.092 for *APOE* (rs429358), and 0.627 for *ABCB1* (rs1045642). In **Table 3**, it is possible to see that the MAF of *VKORC1* (rs9923231) is similar to that of the Colombian population, slightly higher than those of Spain, Puerto Rico, and Europe, higher than the African-American, and lower than East-Asian populations. *VKORC1* (rs7294) MAF is similar to Spain, Puerto Rico, Colombia, and European populations, lower than the African-American, and higher than East Asia. CYP2C9*2 (rs1799853) MAF is slightly lower than Spain, Puerto Rico, Colombia, and Europe and higher than African-American and East Asians. CYP2C9*3 (rs1057910) MAF is similar to Puerto Rico and East Asia, lower than Spain, Colombia, and Europe and higher than African-Americans; *CYP4F2* (rs2108622) MAF is similar to Puerto Rico, Colombia, Europe, and East Asia but lower than Spain and higher than African-Americans. *ABCB1* (rs1045642) MAF is higher than all the populations described but near to the European MAF. *GGCx* (rs11676382) MAF is similar to Puerto Rico and Colombia, lower than Spain and Europe, and higher than African-American and East Asian. Finally, *APOE* (rs429358) MAF is similar to Puerto Rico and East Asia and lower than Spain, Colombia, African-American, and European.

**Table 3:** Comparison of minor allele frequencies obtained in this study and those conducted among in Spain, Puerto Rico, Brazil, Colombia, and African American population (http://www.ensembl.org/Homo_sapiens/Info/Index; modified from Visser et al., 2005; Kocael et al., 2019).

Polymorphic variant	Chile (this study)	Spain	Puerto Rico	Colombia	African-American	European	East Asia	Effect on VKA dose
*VKORC1 (rs9923231)*	0.467	0.360	0.389	0.420	0.054	0.388	0.885	Decrease
*VKORC1 (rs7294)*	0.311	0.355	0.341	0.356	0.454	0.366	0.112	Increase
CYP2C9*2 (rs1799853)	0.081	0.140	0.139	0.122	0.008	0.124	0.001	Decrease
CYP2C9*3 (rs1057910)	0.041	0.084	0.043	0.064	0.002	0.073	0.034	Decrease
*CYP4F2 (rs2108622)*	0.229	0.355	0.288	0.282	0.082	0.290	0.214	Increase
*ABCB1 (rs1045642)*	0.627	0.463	0.428	0.441	0.150	0.518	0.398	Decrease
*GGCx* (*rs11676382*)	0.036	0.093	0.034	0.037	0.002	0.094	0.000	Decrease
*APOE* (*rs429358*)	0.092	0.140	0.106	0.154	0.268	0.155	0.086	Decrease

### Genotype and VKA Dose Ratio in the Study Population

The continuous variable WTD did not have a normal distribution. The logarithm of the WTD was determined to be the optimal transformation for this analysis. The clinical variables that influenced the logarithm of WTD were sex (*p* << 0.0001), age (*p* < 0.0001), BMI (*p* < 0.0002), and INR at the beginning of treatment (*p* < 0.0011). The pharmacogenetic variables, that is, the polymorphisms analyzed which influenced the logarithm of the WTD, were CYP2C9*2 (rs1799853) (*p* < 0.0342), CYP2C9*3 (rs1057910) (*p* < 0.0001), and *VKORC1* (rs9923231) (*p* < 0.0001).

### Algorithm for Acenocoumarol Dosing in the Chilean Population

After the clinical and pharmacogenetic variables that influenced the logarithm of WTD were selected, a linear regression was performed using only the clinical variables, resulting in a model with an *R*^2^ of 0.2013, adjusted *R*^2^ of 0.19, and model *p*-value of <0.0001. A linear regression was then performed using only the pharmacogenetic variables *VKORC1* (*rs9923231*), CYP2C9*2 (*rs1799853*), and CYP2C9*3 (*rs1057910*), producing a model with a *R*^2^ of 0.3790, adjusted *R*^2^ of 0.3685, and model *p*-value <0.0001. Finally, a linear regression was performed using the genetic factors *VKORC1* (*rs9923231*), CYP2C9*2 (*rs1799853*), and CYP2C9*3 (*rs1057910*) and the clinical variables age, sex, BMI, and initial INR to produce a single model explaining the variability in the logarithm of acenocoumarol WTD, with *R*^2^ of 0.5147, adjusted *R*^2^ of 0.4999, and model *p*-value <0.0001 (**Table 4**). Therefore, the algorithm equation developed in this study is the following:

**Table 4:** Linear regression including genetic factors and clinical variables in a single model.

				*N* observed	287
				Model *p*-value*	< 0.0001
				*R* ^2^	0.5147
				Adjusted *R*^2^	0.4999
Variable	Coefficient	Standard error	*p*-value*	CI (95%)	
Sex (men)	0.1668786	0.0407027	0.000	0.0867528	0.2470045
Age	-0.008101	0.001472	0.000	-0.0109987	-0.0052034
Initial INR	-0.0547186	0.0168253	0.001	-0.0878404	-0.0215969
BMI	0.0125554	0.0035861	0.001	0.0054959	0.0196149
CYP2C9*2 (***rs1799853***)					
*1/*2	-0.1067491	0.0538426	0.048	-0.2127418	-0.0007565
CYP2C9*3 (***rs1057910***)					
*1/*3	-0.3227895	0.0806461	0.000	-0.4815465	-0.1640324
*3/*3	-0.7465348	0.2416193	0.002	-1.222178	-0.2708915
***VKORC1*** (***rs9923231***)					
*G/A*	-0.2704925	0.0479039	0.000	-0.3647945	-0.1761906
*A/A*	-0.7008277	0.0583063	0.000	-0.8156074	-0.586048
Constant	3.080551	0.1622701	0.000	2.761112	3.33999

^*^P < 0.05 is considered as statistically significant.

BMI, body mass index; INR, international normalized ratio.

Log WTD = 3.081 + (0.167 × men) - (age × 0.0081) - (initial INR × 0.055) + (BMI × 0.013) - (CYP2C9*1/*2 × 0.107) - (CYP2C9*1/*3 × 0.323) - (CYP2C9*3/*3 × 0.746) - (VKORC1 G/A × 0.270) - (VKORC1 A/A × 0.701).

## Discussion

The patients enrolled in this study are a representative sample of the Chilean population, which is predominantly Amerindian–Caucasian admixture (9.8% of this sample) (Acuña et al., 2000). The group had an average BMI of 29.2 and median BMI of 28.4, classifying these patients as overweight (BMI 25–29.9). These data are consistent with the results of the most recent National Health Survey 2016–2017 (NHS, 2018), which indicated that 39.8% of the population was overweight (43.3% of males and 36.4% of females) and that among Chilean people in the age range of our study patients (65 years or older), 41.2% were overweight and 34.5% were obese. Notably, the time to reach therapeutic range in these patients was 308 ± 343 days on average, with a median of 206 days (**Table 2**). The patients are at high risk for stroke or hemorrhage while out of the therapeutic range. As relying exclusively on INR for dose adjustment is known to delay the time to reach the therapeutic range, the proposed pharmacogenetic dosage algorithm might be quite useful for the Chilean population.

As noted above, other published algorithms differ in the number of variables included and the weight of these variables, as well as in the type of population and methods used to develop the predictive model. The clinical variables included in these algorithms differ essentially in terms of inclusion of non-genetic variables such as sex, BMI, and use of amiodarone or enzyme-inducer drugs. In terms of the genetic variants, all algorithms published to date have included *CYP2C9* and *VKORC1* polymorphisms, whereas *CYP4F2* and *ApoE* are used only in some models. In addition, several models have been designed exclusively for patients with deep vein thrombosis and/or pulmonary embolism (Borobia et al., 2012), while others have included patient cohorts with phenprocoumon and acenocoumarol treatment. The genetic variables included CYP2C9 and VKORC1 genes and the clinical variables include weight, height, sex, age, and amiodarone use and explained up to 76% of stable dose (van Schie et al., 2011). Another algorithm for acenocoumarol included clinical factors (age, body mass index, and body surface area) and genetic variants (VKORC1, CYP2C9*, and CYP4F2 polymorphisms) and explained up to 50% of stable dose (Cerezo-Manchado et al., 2013).

The original Clinical Pharmacogenetics Implementation Consortium (CPIC) algorithm published in the United States accounted for 47% of warfarin dose variability and included the clinical variables age, amiodarone use, weight, height, use of CYP2C9 inducers, and race/ethnicity as well as the pharmacogenetic factors *VKORC1* (*rs9923231*), CYP2C9*2 (*rs1799853*), and CYP2C9*3 (*rs1057910*). CPIC suggests using CYP2C9*5 (*rs28371686*) if the patient is African-American and added *CYP4F2* as an optional factor in the most recent update in 2017 (**Table 4**) (Johnson et al., 2017). Three algorithms have been published for Latin American populations. The algorithm for the population of Puerto Rico (Ramos et al., 2012), which explained 51% of the variability in warfarin dosage, was performed only in men, includes non-genetic variables such as age, initial INR, and use of amiodarone and the genetic variables *VKORC1* (*rs9923231*), CYP2C9*2 (*rs1799853*), CYP2C9*3 (*rs1057910*), and CYP2C9*5 (*rs28371686*). The CYP2C9*5 (*rs28371686*) polymorphism was included due to the presence of a large African-American component in this population (**Table 5**). The Brazilian algorithm, in turn, accounted for 40% of the variability in warfarin dosage and includes the non-genetic variables age, sex, use of amiodarone or CYP2C9 inducers, and self-declared race, which, according to the Brazilian census criteria, includes white, mixed race, or black. The genetic variables included were *VKORC1* (*rs9923231*), CYP2C9*2 (*rs1799853*), and CYP2C9*3 (*rs1057910*) (Botton et al., 2011; Santos et al., 2015), similar to our model. Finally, the Colombian model explained 45.9% of the variability in warfarin dosage. This model included non-genetic variables (age, use of amiodarone, weight, height, use of CYP2C9 inducers, and race/ethnicity), and the genetic variables were the same as those in our model (**Table 4**) (Galvez et al., 2018).

**Table 5:** Comparison between the present study and the vitamin K antagonists (VKA) dosage algorithms published for other populations.

Algorithm		Chile (this study)	Spain (Borobia et al., 2012)	Germany (van Schie et al, 2011)	CPIC (Johnson et al., 2017)	Puerto Rico (Ramos et al., 2012)	Brazil (Santos et al., 2015)	Colombia (Galvez et al., 2018)
Drug	VKA	Acenocoumarol	Acenocoumarol	Acenocoumarol	Warfarin	Warfarin	Warfarin	Warfarin
Clinical variables	Age	X	X	X	X	X	X	X
	Sex	X		X			X	
	Initial INR	X				X		
	Amiodarone use		X		X	X	X	X
	Weight			X	X		X	X
	Height			X	X			X
	Body mass index	X	X					
	CYP2C9 inducer use		X	X	X		X	X
	Race/ethnicity				X		X	X
	% contribution to the final model	19%	22%	23.7%	N.D	19%	N.D	15.9%
Genetic variables	*VKORC1* (*rs9923231*)	X	X	X	X	X	X	X
	CYP2C9*2 (*rs1799853*)	X	X	X	X	X	X	X
	CYP2C9*3 (*rs1057910*)	X	X	X	X	X	X	X
	CYP2C9*5 (*rs28371686*)				African-Americans	X		
	*ApoE (rs429358)*		X					
	*CYP4F2* (*rs2108622*)		X		Optional			
	% contribution to the final model	36,9%	39%	52.6%	N.D	32%	N.D	30%
Percentage of variability in VKA dosage explained		49.99%	60.6%	76.3%	47%	51%	40%	45.9%

N.D., no data published.

All of the above models were developed for warfarin. In Chile, however, as in Spain, the Ministry of Health indicates that acenocoumarol should be used in preference to any other coumarin. The Spanish model included the non-genetic variables sex, age, BMI, and initial INR value, and the genetic variables were the *VKORC1* (*rs9923231*), CYP2C9*2 (*rs1799853*), and CYP2C9*3 (*rs1057910*) polymorphisms. The model explained 60% of the variability in acenocoumarol dosage, similar to our results (49.99%). Our model showed no association between WTD and *CYP4F2* or *ApoE* polymorphisms, which are included in the Spanish study (Borobia et al., 2012). Therefore, the final algorithm for this Amerindian-Caucasian admixture includes the genetic factors *VKORC1* (*rs9923231*), CYP2C9*2 (*rs1799853*), and CYP2C9*3 (*rs1057910*) and non-genetic variables age, sex, BMI, and initial INR, explaining almost 50% of the variability in WTD in the Chilean population studied. The algorithm equation developed for the group of Chilean patients explains a similar percentage of dose variability as the Puerto Rican, Brazilian, and Colombian algorithms (**Table 5**).

There are a number of limitations in this study. A number of parameters that affect coumarin dosage were not included, such as smoking status and use of other concomitant medications. These are important factors to keep in mind when establishing a stabilized dosage of acenocoumarol. Moreover, as this is a discovery cohort (also called a derivation or retrospective cohort), the next step is to perform a clinical application of this algorithm in a well-designed prospective validation cohort (also called a test cohort) to obtain sensitivity, specificity, and, of course, predictive values (Tong et al., 2016) before the algorithm is used routinely for acenocoumarol dose adjustment in Chilean patients.

## Conclusion

Establishing appropriate coumarin dosage is challenging due in part to significant inter-individual variability in the dose required to achieve a stable range of anticoagulation (INR 2.0–3.0). Various genetic and non-genetic factors have been associated with coumarin dosage requirements, and pharmacogenetic-guided dosing algorithms for warfarin and acenocoumarol have been developed for mixed populations with different predictive values. Here, we have developed the first acenocoumarol dosage algorithm for this Chilean mixed Amerindian–Caucasian population, which explains about 50% of dose variability. After clinical validation, this algorithm could provide a new tool for adjusting VKA dosage, considerably improving TTR, and thereby reducing thrombotic and hemorrhagic risks in Chilean patients.

## Data Availability Statement

The original contributions presented in the study are included in the article/supplementary files, further inquiries can be directed to the corresponding author.

## Ethics Statement

The research was authorized by the Ethics Committees of the University of Chile Faculty of Medicine, Project 222-2015, and the WMHS, Protocol No. 027/2016. The patients/participants provided their written informed consent to participate in this study.

## Author Contributions

AR participated in the conception of the research, analysis of data, and writing of the manuscript. EN participated in the conception of the research and writing of the manuscript. MS, MR, GV, and FT conducted the experimental analyses. MB, AA, DC, JM, GB, PS, FM, GG, and PV facilitated the enrolment of patients. LQ participated in the conception of the research, the analysis of data, and the writing of the manuscript.

## Funding

The authors declare that this study received funding from Roche Diagnostics Chile (Probes Donation). The funder was not involved in the study design, collection, analysis, interpretation of data, the writing of this article or the decision to submit it for publication.

## Conflict of Interest

The authors declare that the research was conducted in the absence of any commercial or financial relationships that could be construed as a potential conflict of interest.
