# Corrigendum: A pharmacogenetically-guided acenocoumarol dosing algorithm for Chilean patients: a discovery cohort study

**DOI:** 10.3389/fphar.2025.1588440

**Published:** 2025-03-31

**Authors:** Angela Roco, Elena Nieto, Marcelo Suárez, Mario Rojo, Maria Paz Bertoglia, Gabriel Verón, Francisca Tamayo, Annabella Arredondo, Daniela Cruz, Jessica Muñoz, Gabriela Bravo, Patricio Salas, Fanny Mejías, Gerald Godoy, Paulo Véliz, Luis Abel Quiñones

**Affiliations:** ^1^ Laboratory of Chemical Carcinogenesis and Pharmacogenetics, Department of Basic and Clinical Oncology, Faculty of Medicine, University of Chile, Santiago, Chile; ^2^ Escuela de Bioquímica Facultad de Ciencias de la Vida, Universidad Andrés Bello, Santiago, Chile; ^3^ Western Metropolitan Health Service, Santiago, Chile; ^4^ San Juan de Dios Hospital, Santiago, Chile; ^5^ Latin American Network for Implementation and Validation of Clinical Pharmacogenomics Guidelines (RELIVAF-CYTED), Madrid, Spain; ^6^ Institute of Population Health, University of Chile, Santiago, Chile; ^7^ Instituto de Salud Pública, Universidad Andrés Bello, Santiago, Chile; ^8^ Curacaví Hospital, Curacaví, Chile; ^9^ Dr. Salvador Allende G. Reference Health Center, Santiago, Chile; ^10^ San José de Melipilla Hospital, Melipilla, Chile

**Keywords:** acenocoumarol, coumarins, algorithm, pharmacogenetics, pharmacogenomics, anticoagulation

In the published article, there was an error in Figure 1 as published. The patient numbers are listed as 377 --> 377 --> 304, but it should say 377 --> 305 --> 304. The corrected Figure 1 and its caption appear below.

**FIGURE 1:**
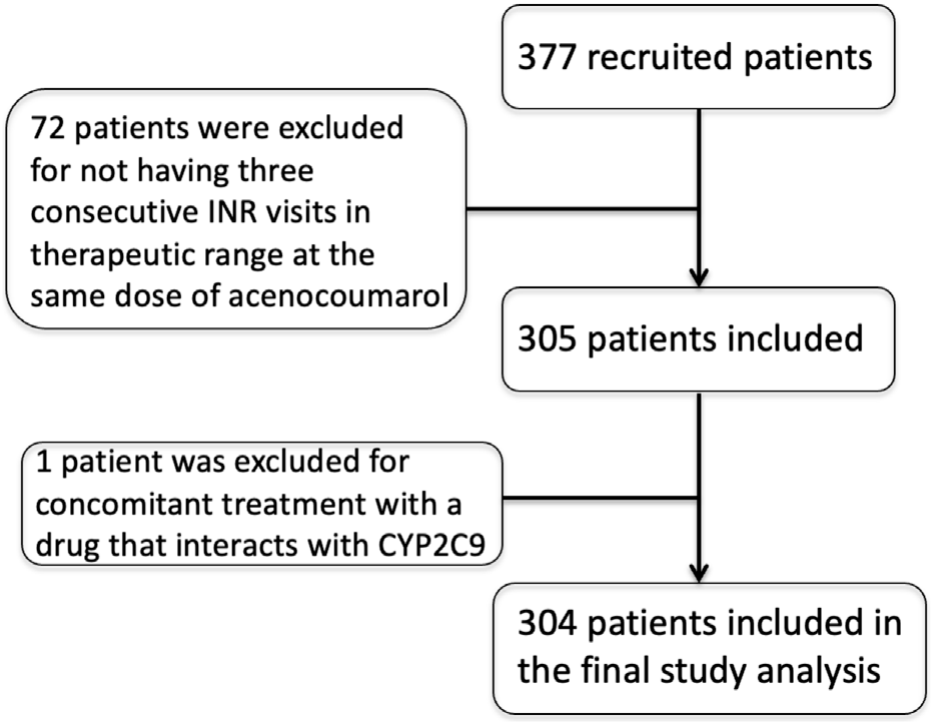
Patient recruitment and selection flowchart.

In the published article, there was an error in **Results,** *Algorithm for Acenocoumarol Dosing in the Chilean Population*. In the formula two factors have decimals placed incorrectly. It’s a formatting error, but it is significant as it impacts the dose calculations.

The formula previously stated:

“Log WTD = 3.081 + (0.167 × men) - (age × 0.081) - (initial INR × 0.55) + (BMI × 0.013) - (CYP2C9*1/*2 × 0.107) - (CYP2C9*1/*3 × 0.323) - (CYP2C9*3/*3 × 0.746) - (VKORC1 G/A × 0.270) - (VKORC1 A/A × 0.701).”

The corrected formula appears below:

“Log WTD = 3.081 + (0.167 × men) - (age × 0.0081) - (initial INR × 0.055) + (BMI × 0.013) - (CYP2C9*1/*2 × 0.107) - (CYP2C9*1/*3 × 0.323) - (CYP2C9*3/*3 × 0.746) - (VKORC1 G/A × 0.270) - (VKORC1 A/A × 0.701).”

In the published article, there was an error in **Results,** *Genotype Distribution in the Study Population,* paragraph 1.

This sentence previously stated:

“The analysis of the HWE showed that only CYP2C9*3 (rs1057910) is in HWE (chi2 = 4.67). All other SNPs, VKORC1 (rs9923231) (chi2 = 0.09), VKORC1 (rs7294) (chi2 = 0.62), GGCx (rs11676382) (chi2 = 1.2), CYP4F2 (rs2108622) (chi2 = 1.02), ApoE (rs429358) (chi2 = 0.08), ABCB1 (rs1045642) (chi2 = 0.68), and CYP2C9*2 (rs1799853) (chi2 = 2.33), are not in HWE.”

The corrected sentence appears below:

“The analysis of the HWE showed that only CYP2C9*3 (rs1057910) is not in HWE (chi2 = 4.67). All other SNPs, *VKORC1*(rs9923231) (chi2 = 0.09), *VKORC1* (rs7294) (chi2 = 0.62), GGCx (rs11676382) (chi2 = 1.2), *CYP4F2* (rs2108622) (chi2 = 1.02), *ApoE* (rs429358) (chi2 = 0.08), *ABCB1* (rs1045642) (chi2 = 0.68), and CYP2C9*2 (rs1799853) (chi2 = 2.33), are in HWE.”

The correspondence email for author Luis Abel Quiñones has been updated in the published article to lquinone@uchile.cl.

The authors apologize for these errors and state that this does not change the scientific conclusions of the article in any way. The original article has been updated.

